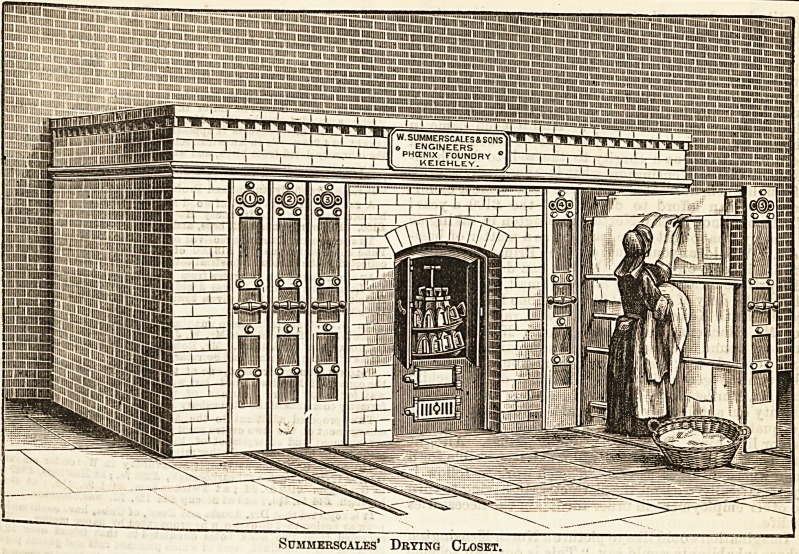# The Laundry

**Published:** 1892-10-01

**Authors:** 


### PRACTICAL DEPARTMENTS.

### III.—THE LAUNDRY (*concluded from page* 414, *Vol. XII.*)

Many manufacturers' catalogues contain a plan, sketch, or description of what a "complete laundry" should be; and if we propose to construct and fit up a new one, from the beginning, we find our ideas enlarged and our suggestions practically facilitated by these aids. However, we do not need to build very often, and if we wished to do so, lack of funds would probably stand in our way. When an institution

Summerscales' Mangle.

possesses a laundry, it generally inclines to make the best of it, enlarging or improving from time to time and adding such new machines as are necessitated by the wearing out of the old ones. In this way the work goes on satisfactorily, and modern improvements gradually gain admittance into the old as well as the new establishments. Laundry machinery seems costly when we consider the price of each separate appliance, but our ideas change when we learn the number of years which pass by without any change or repair being required, owing to the strength and durability of the plant. The mangle, which is an essential in laundry work, may surely be described as a very "long-lived" appliance, and there are many good varieties of it to be seen in different institutions.

The one shown here is made by Messrs. Summerscales, of Keighley, and is known as a "box-mangle, for hand power, fitted with fly-wheel and handle"; the framework and box are of birch wood, and the beds of Sabien mahogany. A similar but larger mangle is fitted for steam power by the same manufacturers.

The "Ironer," called by some firms the "Steam Calender," is a valuable invention—it does its work beautifully, and not only can a large table cloth be perfectly glazed on both sides and turned out free from the smallest crease, but curtains, and even pocket handkerchiefs, can be rapidly treated by the same huge roller. This machine also serves for airing linen, which is by no means the least of its merits, for every housewife knows the inconvenience of receiving a basket of damp clothes from the laundress, and she must welcome this facility for rendering linen safe for immediate wear. There are steam ironers of all sizes, from the huge calender which makes light of dealing with a big table cloth, down to the small machine which is used for shirt-fronts, collars, or cuffs. All "Ironers" can, however, be trusted with the most delicate lace, and casualties are of infrequent occurrence. Gas can be used instead of steam for heating the calender, but the latter is probably the best, wherever obtainable, for it ensures an even temperature, and allows no possibility of scorching.

Stoves for heating the ordinary irons are of a great many sizes and shapes, and certainly much ingenuity has been applied in their construction. In the various rinsings, blueings, and starchings which clothes undergo there is help to be had from machinery, partial or full, and naturally in hospitals, where all labour has to be paid for, there is more demand for mechanical assistance than in the workhouses and other institutions where employment for the inmates is desirable. In the latter cases, laundry work is not looked upon as objectionable, and we find elderly men and women well content to spend their time at that which has visible results. Labour set merely as task-work must be always uncongenial when compared with that which is evidently useful, of practical benefit to the institutional community which has come to represent "the world" to the dwellers within the walls. It is a digression, perhaps, to say here that we mention "elderly" persons designedly. They are by far the more industrious class of paupers, which points out a truth worth our consideration. If an old person is driven into the workhouse by misfortune, sickness, or whatnot, that individual seldom sits down and bemoans his or her misfortunes, but makes an attempt to do something, and often does it so faithfully that it is soon looked upon as quite natural for "old Kate" or "old Joe" to undertake some little bit of the regular routine, which is a source of untold comfort to that self-respecting personage. It is the young people who are idle—"idle and impudent"—and the mangling or wringing done by their unwilling hands is as slow as the machines will permit it to be, whilst the "hand work" accomplished during the day is generally of a kind and quality to fully explain the reasons of the young person becoming pauperised at so early an age.

At St. Olave's workhouse a neat little machine is used for the goffering of the women's cap borders, and this is worked by the agency of certain steam which would otherwise be wasted. The old lady who does the most responsible parts is very deft at it, and her assistant appeared anxious to do equal justice to the caps of her companions.

Drying closets form a very important conclusion to the treatment undergone by our clothes, and Mr. Taylor is strongly in favour of these being well-lighted as well as

Summerscales' Drying Closet.

heated, which, of course, facilitates the attendant's work. Where possible, it is a great advantage to be able to see all that is going on, but it cannot always be done in laundries of old construction.

The "airing horse" is contrived to move with so little trouble that it takes a very slight exertion of strength to push in or out one which is completely loaded. We conclude with a sketch of an airing closet designed by Messrs. Summerscales.

## Figures and Tables

**Figure f1:**
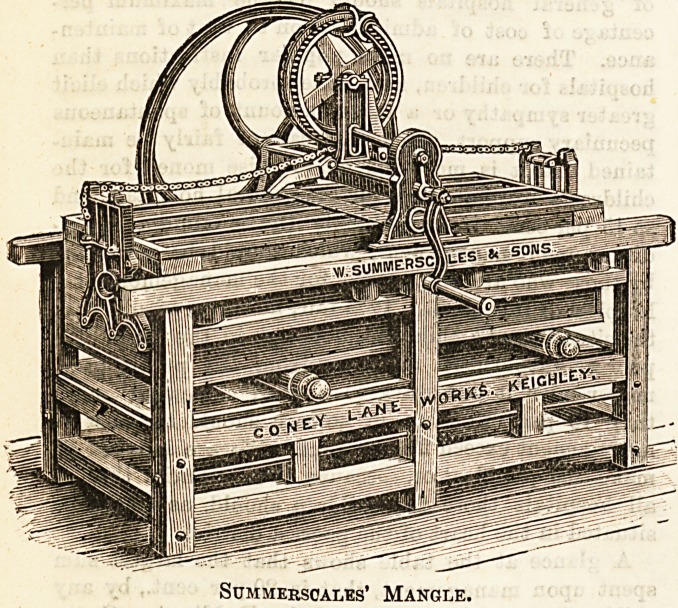


**Figure f2:**